# Cross-cultural adaptation and validation of the Thai version of the functional index for hand osteoarthritis (FIHOA)

**DOI:** 10.1186/s12891-022-05528-x

**Published:** 2022-06-16

**Authors:** Sitthiphong Suwannaphisit, Boonsin Tangtrakulwanich, Porames Suwanno, Nitiphoom Sinnathakorn, Emmanuel Maheu

**Affiliations:** 1 Department of Orthopedics, Faculty of Medicine, Prince of Songkla University, 15 Karnjanavanich Road, Hat Yai, Songkhla, 90110 Thailand; 2 Department of Rheumatology, APHP, Saint-Antoine Hospital, 283, Boulevard Voltaire, Paris, France

**Keywords:** FIHOA, Osteoarthritis, Hand, Thai translation, Psychometric properties, Patient health questionnaires, Validation study

## Abstract

**Background:**

The Functional Index for Hand Osteoarthritis (FIHOA) is a simple tool to assess functional impairment for hand OA patients. The purpose of this study was to translate the FIHOA into the Thai language, and validate it in Thai hand OA patients.

**Methods:**

The FIHOA was translated into Thai (T-FIHOA) according to the principles of cross-cultural adaptation and administered to 102 hand OA patients recruited between September 2020 and July 2021 together with the modified Health Assessment Questionnaire (mHAQ), Disabilities of the Arm, Shoulder and Hand (DASH), and visual analogue scale (VAS) for hand pain. Spearman’s correlation coefficient and intraclass correlation coefficient (ICC) were used to check the test-retest reliability of each item and the total scores in the translated questionnaire. The internal consistency reliability was evaluated using Cronbach’s alpha. The external construct validity was assessed using correlations between the T-FIHOA, mHAQ, DASH and hand pain VAS.

**Results:**

The T-FIHOA had a unidimensional structure. The ICC was > 0.9 and the Cronbach’s alpha of 0.92 indicated excellent reliability and internal consistency, respectively. The external validity tests indicated moderate correlation with the hand pain VAS (r = 0.37, *P* <  0.01) and moderate to strong correlations with the mHAQ (r = 0.63, *P* <  0.01), and DASH score (r = 0.52, *P* <  0.01). The T-FIHOA had the highest effect size (ES) and standardized response mean (SRM) (− 0.37 and − 0.58 respectively) among all questionnaires except for the VAS when assessing changes between baseline and week 4.

**Conclusions:**

The T-FIHOA is a good and reliable assessment tool freely available for practitioners/researchers to evaluate functional impairment in Thai hand OA patients.

## Introduction

Osteoarthritis (OA) is characterized by joint pain, often associated with use, and impaired joint function. Osteoarthritis can affect any joint, most commonly the hand, knee, spine, and hip. In the hand, the distal interphalangeal joint, proximal interphalangeal joint, and first carpometacarpal joint are the most commonly affected [1]. In the elderly population, the prevalence of radiographic hand OA can reach 80% [2]. Symptomatic OA of the hand can lead to functional impairment due to pain, stiffness, deformity, and loss of strength, limiting the individual’s ability to perform daily tasks [2–5]. OA of the hand usually progresses slowly in terms of articular damage and functional hand impairment [6]. When a patient is diagnosed with hand OA, an objective assessment of OA of the hand is mandatory and useful to tailor the management to each patient. 

A number of instruments have been developed to measure pain and functional disability in patients presenting with possible OA, ranging from physical assessment by a trained assessor to self-administered hand disability questionnaires. The Arthritis Impact Measurement Scales 2 (AIMS2) [7], the Functional Index for Hand Osteoarthritis (FIHOA) [8–10], the Cochin Hand Functional Disability Scale [11], the Score for the Assessment and Quantification of Chronic Rheumatoid Affections in the Hands (SACRAH) [12], the Multidimensional Health Assessment Questionnaire (MDHAQ) [13], and the Australian/Canadian Osteoarthritis Hand Index (AUSCAN) [14] are commonly used in practice and clinical trials [15, 16] to assess functional impairment in OA of the hand.

 Currently, the FIHOA questionnaire is one of the most popular for assessing physical function in patients with OA of the hand [16]. The Osteoarthritis Research Society International recommends the FIHOA as the preferred tool for use in clinical trials of hand OA as it is not copyrighted and is free to use by researchers and the rheumatology/orthopedic community [4, 17]. It was the first hand OA-specific instrument, developed as a 10-item questionnaire in French, validated by Dreiser and Maheu, then validated in English [8]. Inter-observer reproducibility and sensitivity to change were confirmed in 1997 and 2000 [9]. The FIHOA has been translated into over 23 languages to date and is undergoing cross-cultural adaptation and validation in three additional language versions. The reliability and validity of the FIHOA have already been investigated and published in the English, Dutch, Norwegian, Swedish, Italian, Portuguese, Persian, Korean, Japanese, Turkish, and Arabic languages [8–10, 18–24]. All informations and linguistic versions are publicly available at FIHOA.net.

Although there are Asian versions available as mentioned, neither the Korean [21] nor the Japanese [22] versions are suitable for use in Thailand due to linguistic, and also important cultural and contextual differences. Therefore, the aim of this study was to develop a Thai version of the FIHOA questionnaire (T-FIHOA), and to perform test-retest reliability and concurrent validity of this Thai questionnaire according to the guidelines for the process of Cross-Cultural Adaptation of Self-Report Measures [25].

## Methods

The translation process followed the international guidelines as described by Beaton et al. [24] This method consists of 5 stages: (1) translation, (2) synthesis, (3) back translation, (4) expert committee review with the developers of the questionnaire, and (5) pre-testing. Subsequently, a prospective observational single-center study was undertaken for the overall validation processes, including the assessment of the metrologic properties of the T-FIHOA in Thai hand OA patients.

### Translation and cultural adaptation

An expert committee was initiated including two persons whose Thai was the mother language and who were also fluent in English. Translator 1 was an orthopedist who was familiar with the concepts being examined in the questionnaire. Translator 2 was a staff member of the Liberal Arts Faculty, without any medical or clinical background. Both translators independently translated the questionnaire into Thai. The two versions were assessed by the expert committee and synthesized into a preliminary version of the T-FIHOA. It was then back translated into English independently by two English native professional translators, one with a medical background and one without from the faculty of Liberal Arts, both of whom were blinded to the study aims and FIHOA concepts. After back translations, there still was remaining linguistic and culture problems. In Thai culture, Thai people did not handshake with others. People have to pay respect to the elders or the activities such as prostate together after the chant. To adapt this item in order to keep capturing the activities and function assessed, these problems were resolved through consultation with the developers of the FIHOA (E.M.) by discussion. The T-FIHOA was pretested on 15 Thai hand OA patients to identify potentially difficult words or phrases and a written report submitted to the developers. The final translation of T-FIHOA including cultural adaptation was finalized (Table 1).

**Table 1:** Thai version of the Functional Index for Hand Osteoarthritis

	Original version of FIHOA	Thai version of FIHOA
Question 1	Are you able to turn a key in a lock?	ท่านสามารถไขแม่กุญแจได้หรือไม่
Question 2	Are you able to cut meat with a knife?	ท่านสามารถใช้มีดหั่นเนื้อสัตว์ได้หรือไม่
Question 3	Are you able to cut cloth or paper with a pair of scissors?	ท่านสามารถใช้กรรไกรตัดผ้าหรือกระดาษได้หรือไม่
Question 4	Are you able to lift a full bottle with the hand?	ท่านสามารถยกขวดหนักๆ ด้วยมือข้างเดียวได้หรือไม่
Question 5	Are you able to clench your fist?	ท่านสามารถกำหมัดได้แน่นๆ หรือไม่
Question 6	Are you able to tie a knot?	ท่าสามารถผูกเงื่อนได้หรือไม่
Question 7A	For women – Are you able to sew?	สำหรับสุภาพสตรี – ท่านสามารถเย็บผ้าได้หรือไม่
Question 7B	For men – Are you able to use a screwdriver?	สำหรับสุภาพบุรุษ – ท่านสามารถใช้ไขควงได้หรือไม่
Question 8	Are you able to fasten buttons?	ท่านสามารถติดกระดุมได้หรือไม่
Question 9	Are you able to write for a long period of time (10 min)?	ท่านสามารถเขียนหนังสือเป็นเวลานานได้หรือไม่ (10 นาที)
Question 10	Would you accept a handshake without reluctance?	ท่านสามารถยกมือไหว้ทักทายผู้อื่นได้หรือไม่
Scoring System		
0	Possible without difficulty	สามารถทำได้อย่างอิสระ
1	Possible with slight difficulty	สามารถทำได้ด้วยความยากลำบากเล็กน้อย
2	Possible with importance difficulty	สามารถทำได้ด้วยความยากลำบาก
3	Impossible	ไม่สามารถทำได้

### Validation

#### Participant recruitment

Hand OA patients were recruited at the outpatient Orthopedics department of a single tertiary center in the South of Thailand September 2020 and July 2021. The classification used for the diagnosis of hand OA was the American College of Rheumatology (ACR) classification criteria [26]. The study enrolled new cases or already followed hand OA patients over 18 years old. Patients with secondary hand OA including post-rheumatic diseases or post-traumatic OA were excluded. All participants had received standard treatment for hand OA which was not modified. The participants were divided into two groups: symptomatic and not or very few symptoms using the threshold of > 5 points (defining symptomatic patients) proposed by the FIHOA developers [8] as cut-off to classify patients. A written consent was obtained from each participant after information on this non interventional study. The office of Human Research Ethics Committee of faculty of Medicine, Prince of Songkla University (IRB number: 63–187–11-1) approved the study protocol, performed in accordance with the ethical standards mentioned in the IRB approved study protocol and the Declaration of Helsinki.

#### Data collection

Data collected on the date of enrollment were medical history including hand OA history, duration of hand pain/stiffness, and prior and current treatments. Postero-anterior plain radiographs were obtained to assessing hand OA severity. Patient’s scoring of the T-FIHOA, the Thai version of the modified - Stanford Health Assessment Questionnaire (mHAQ), the visual analogue scale for pain (VAS pain), and the Thai version of the Disabilities of the Arm, Shoulder and Hand (DASH) were collected. We assessed the test-retest reliability at one to 2 weeks’ time-interval using T-FIHOA in patients whose symptoms and treatment remained stable. To perform the retests, the investigator could use different means including a face-to-face visit or a telephone interview depending upon the convenience for participants. In order to measure the responsiveness, the T-FIHOA and DASH questionnaires were assessed at baseline and 6 weeks. Treatment for hand osteoarthritis started after collecting baseline data. Patients received systemic pharmacological treatments as well as education for joint protection and strengthening exercises. If the symptoms were not improved, treatment and dosages could be adapted.

### Questionnaires

#### Thai version of the functional index for hand osteoarthritis (Table 1)

The FIHOA is a 10-item self-administered. The answers are scored according to a 4-point scale, as follow: 0, possible without difficulty; 1, possible with slight difficulty; 2, possible with important difficulty; 3, impossible. The total score ranges from 0 to 30 [8]. In this study, question 10 was modified because in Thai culture we usually don’t handshake when meeting with another person. Therefore, we adapted the question to Thai culture as “Are you able to press your palms together in front of your chest?”. Participants with total scores of 5 or more were defined as having symptomatic hand OA [8].

#### Thai version of the disabilities of the arm, shoulder and hand (DASH)

DASH, Thai version [27], is made of 30 items. Items 1–21 score the level of difficulty when performing various physical activities related to upper limb, shoulder and hand problems; items 22 and 23 evaluate the extent of social activities and work/daily activities limitations; item 29 questions on the impact of upper limb problems on sleep, and item 30 assesses patient’s self-perception with respect to his/her upper limb conditions.

#### Visual analogue scale for pain (VAS pain) global

Participants were asked to score the level of pain intensity on a horizontal scale from “0 = no pain” to “100 = worst pain imaginable.”

#### Thai version of the modified Stanford health assessment questionnaire (mHAQ)

Thai HAQ includes 20 items in eight domains adapted from the original HAQ-DI to suit Thai culture and activities [28]. The ability to perform an activity for each item is rated on a 0–3 scale, from 0 (no difficulty in performing that activity) to 3 (inability to perform that activity). The requirement of a device or physical assistance for any item increases the lower score to 2.

### Statistical analysis

Demographic data of the study population were presented as mean ± standard deviation (SD) for continuous variables and frequency with percentage (%) for categorical variables. The independent Student *t* test was used to compare demographic and clinical characteristics of the patients between the symptomatic and non- or mildly symptomatic hand OA groups. To compare the scores of the T-FIHOA between test and retest, the Wilcoxon’s signed-rank test was used. To assess the validity and reliability of the T-FIHOA we used the Spearman’s correlation coefficient (Spearman’s rho), the intra-class correlation coefficient (ICC), the weighted Kappa, and the Cronbach’s alpha. The internal consistency was assessed by using a factor analysis. All analyses were performed using the R program Version 3.4.5 (R Foundation for Statistical Computing, Austria). For comparisons, a *P* value of < 0.05 was considered significant.

### Test-retest reliability

Spearman’s rho, ICC, and weighted Kappa were used to evaluate the test-retest reliability. ICCs were calculated for the total scores. The two-way mixed single measures test was used for estimating the reliability of each item individually. The Spearman’s rho values range from 0.1–1.0 and are considered as weak (0.1–0.3), moderate (0.31–0.5), or strong (> 0.5) respectively. An ICC ≥0.7 indicates a good reliability at the scale level.

### Internal consistency

The internal consistency (i.e., the overall correlation between the items within a scale) was assessed by calculating Cronbach’s alpha. If the value was > 0.7 the internal consistency was considered acceptable to good.

### Internal structure and external validity

Factor analysis was used to evaluate the internal construct validity while external validity was examined by assessing the correlations between the T-FIHOA and the Thai version of the mHAQ, and the pain VAS using Spearman’s correlation coefficient.

## Results

### Demographics and clinical characteristics

One hundred and two patients answered the T-FIHOA. All patients completed the questionnaires. The demographic characteristics of the participants are presented in Table 2. Mean age was 65.9 years, 74.5% were females. The mean T-FIHOA score was 5.9 (5.5). The one hundred and two hand OA patients were analyzed equally divided into a symptomatic versus non-or mildly symptomatic group.

**Table 2:** Patient demographics and clinical characteristics

Variable	Total population (*N* = 102)	Symptomatic hand OA group (*N* = 51)	Non−/mildly symptomatic hand OA group (*N* = 51)	*P*-Value
Age (Year)	65.6 (±8.9)	64.6 (±7.7)	66.6 (±9.9)	0.257
Female gender	78 (74.5%)	40 (78.4%)	38 (76.5%)	0.815
Body mass index (kg/m^2^)	24.4 (3.6)	23.9 (3.2)	24.8 (3.9)	0.205
Disease duration, months	29.4 (24.4)	25.3 (23.2)	33.4 (25.1)	0.09
T-FIHOA score, 0–30	6 (5.5)	7.5 (6.2)	4.4 (4.3)	0.004
mHAQ, 0–3	0.8 (0.7)	1 (0.8)	0.7 (0.6)	0.058
DASH score, 0–100	26.8 (20.9)	29.9 (22.4)	23.8 (19.2)	0.145
Hand pain VAS, 0–100	36.4 (30.3)	63.7 (14.7)	9 (10.6)	< 0.001
Number of radiogically affected joints^a^, (KL grade ≥2)	4.1 (2.8)	5.1 (3.2)	3.1 (1.9)	< 0.001
Right hand dominant				

Values are given as mean ± standard deviation or frequency (percentage)

*OA* Osteoarthritis, *T-FIHOA* The Thailand version of the Functional Index of Hand Osteoarthritis, *mHAQ* Modified Health Assessment Questionnaire, *VAS* Visual analogue scale, *KL grade* Kellgren-Lawrence grade

Sixteen joints for each hand, including five distal interphalangeal joints, four proximal interphalangeal joints, five metacarpophalangeal joints and the base of the thumb joints were evaluated for the presence of osteophytes, joint space narrowing, sclerosis and cysts. Each joint was graded using a modified K-L grade 0–4

*P value < 0.05

The mean T-FIHOA score was obviously higher in the symptomatic hand OA group than in the non/mildly symptomatic group (7.5 ± 6.2 vs 4.4 ± 4.3, *P* <  0.01). The mean mHAQ score was also higher in the symptomatic hand OA group (1 ± 0.8) than in the non/mildly symptomatic group (0.7 ± 0.6, *P* = 0.06). There were no statistically significant differences between the two groups with regard to demographic or baseline clinical characteristics, including age (64.6 ± 7.7 vs 66.6 ± 9.9, *P* = 0.26), disease duration (25.3 ± 23.2 vs 33.4 ± 25.1, *P* = 0.09), proportion of women (40 (78.4%) vs 38 (76.5%), *P* = 0.82), body mass index (23.9 ± 3.2 vs 24.8 ± 3.9, *P* = 0.21), and DASH score (29.9 ± 22.4 vs 23.8 ± 19.2, *P* = 0.15) except for the number of radiologically affected joints (5.1 ± 3.2 vs 3.1 ± 1.9, *P* <  0.01). and pain score (63.7 ± 14.7 vs 9.0 ± 10.6, *P* <  0.01) which were higher in the symptomatic group.

### Test-retest reliability

The patients completed the T-FIHOA twice at a 7–14 days intervals. Treatments between two assessments remained stable. Table 3 shows T-FIHOA total and item by item scores during the test-retest exercise. Mean total scores of the T-FIHOA were 5.97 (SD = 5.52) and 5.41 (SD = 5.23) at the initial assessments and 2-week follow-up respectively (Wilcoxon signed rank test, *P* = 0.60). Spearman’s rho value was 0.99 for the T-FIHOA total score; Spearman’s rho ranged between 0.87 and 0.99 for each item and for the global score. ICC were excellent both for the total score (ICC = 0.99), and for each single item (range, 0.94–0.99).

**Table 3:** Test-retest reliability of the T-FIHOA

T-FIHOA test – T-FIHOA retest	Test	Retest	Spearman’s rho^a^	ICC	95% CI
Item 1 – Item 1 retest	0.48 (±0.66)	0.46 (±0.61)	0.97	0.97	−0.02 – 0.05
Item 2 – Item 2 retest	0.64 (±0.69)	0.59 (±0.67)	0.92	0.96	0.00–0.10
Item 3 – Item 3 retest	0.62 (±0.73)	0.55 (±0.65)	0.95	0.96	0.02–0.12
Item 4 – Item 4 retest	0.71 (±0.80)	0.64 (±0.72)	0.97	0.97	0.02–0.12
Item 5 – Item 5 retest	0.64 (±0.64)	0.54 (±0.62)	0.87	0.94	0.04–0.16
Item 6 – Item 6 retest	0.61 (±0.73)	0.57 (±0.71)	0.94	0.97	−0.01 – 0.09
Item 7 – Item 7 retest	0.75 (±0.74)	0.69 (±0.69)	0.95	0.97	0.01–0.11
Item 8 – Item 8 retest	0.40 (±0.60)	0.39 (±0.60)	0.94	0.97	−0.02 – 0.04
Item 9 – Item 9 retest	0.86 (±0.80)	0.73 (±0.70)	0.91	0.94	0.07–0.21
Item 10 – Item 10 retest	0.28 (±0.55)	0.27 (±0.53)	0.99	0.99	−0.01 – 0.03
T-FIHOA total score	5.97 (±5.52)	5.41 (±5.23)	0.99	0.99	0.43–0.69

*T-FIHOA* The Thailand version of the Functional Index of Hand Osteoarthritis, *ICC* Intra-class correlation coefficient, *CI* Confidence interval. Value are given as mean ± standard deviation

^a^Spearman’s rho indicates Spearman’s correlation coefficient

### Internal consistency

Internal consistency was excellent with a Cronbach alpha value of 0.93. When deleting 1 item after another, Cronbach’values remained high, ranging from 0.92 to 0.93. Each individual items of the T-FIHOA was strongly correlated to the total score. All correlations were statistically significant (*P* <  0.01), as shown in Table 4.

**Table 4:** Internal consistency of the T-FIHOA

Items	Mean (SD)	Scale mean if Item is deleted	Scale variance if item is deleted	Adjusted total item Correlation Spearman’s rho^a^	Cronbach’s alpha if item is deleted^b^
Item 1	0.48 (0.66)	4.1	16	0.83	0.92
Item 2	0.64 (0.69)	4.0	15.21	0.87	0.92
Item 3	0.62 (0.73)	3.9	15.21	0.81	0.92
Item 4	0.71 (0.80)	3.9	16	0.77	0.92
Item 5	0.64 (0.64)	4	16.81	0.64	0.93
Item 6	0.61 (0.73)	3.9	15.21	0.79	0.92
Item 7	0.75 (0.74)	3.9	15.21	0.75	0.92
Item 8	0.40 (0.60)	4.1	16.81	0.75	0.92
Item 9	0.86 (0.80)	3.7	16	0.60	0.93
Item 10	0.28 (0.55)	4.3	18.49	0.62	0.93

*T-FIHOA* the Thailand version of the Functional Index of Hand Osteoarthritis

Values are given as mean ± standard deviation or range

^a^Spearman’s rho indicates Spearman’s correlation coefficient

^b^Overall Cronbach’s alpha values for all 10 items are 0.93

### Internal construct validity

**Fig. 1:**
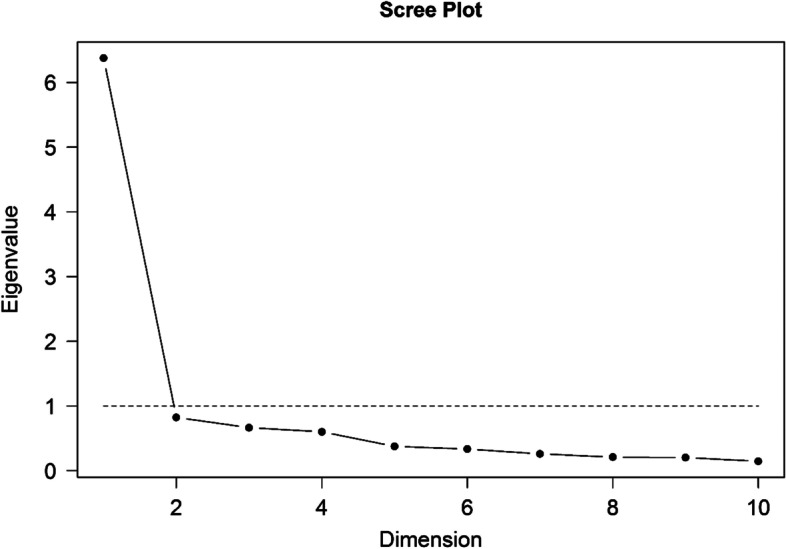
Scree plot of T-FIHOA. The eigenvalue for the first factor was greater than one and accounted for 42% of the total variance. The single elbow in the scree plot also indicated that the T-FIHOA was a unidimensional scale

The internal structural validity of the T-FIHOA was evaluated by a factor analysis. The sample size was adequate with a Kaiser-Meyer-Olkin value of 0.88. The factor model was appropriate with a χ2 value (produced by Bartlett’s test of sphericity) of 441.26 (*P* <  0.01). The eigenvalue of the first factor was 6.37, explaining 42% of the global variance of the T-FIHOA, whilst, the second factor accounted for 24% of the variance. The scree plot had a single elbow curve (Fig. 1). The results from this figure confirmed that the T-FIHOA has a unidimensional structure. The factor loadings, representing associations between each item and the factor, were examined and ranged from 0.52 to 0.92.

### External construct validity

**Fig. 2:**
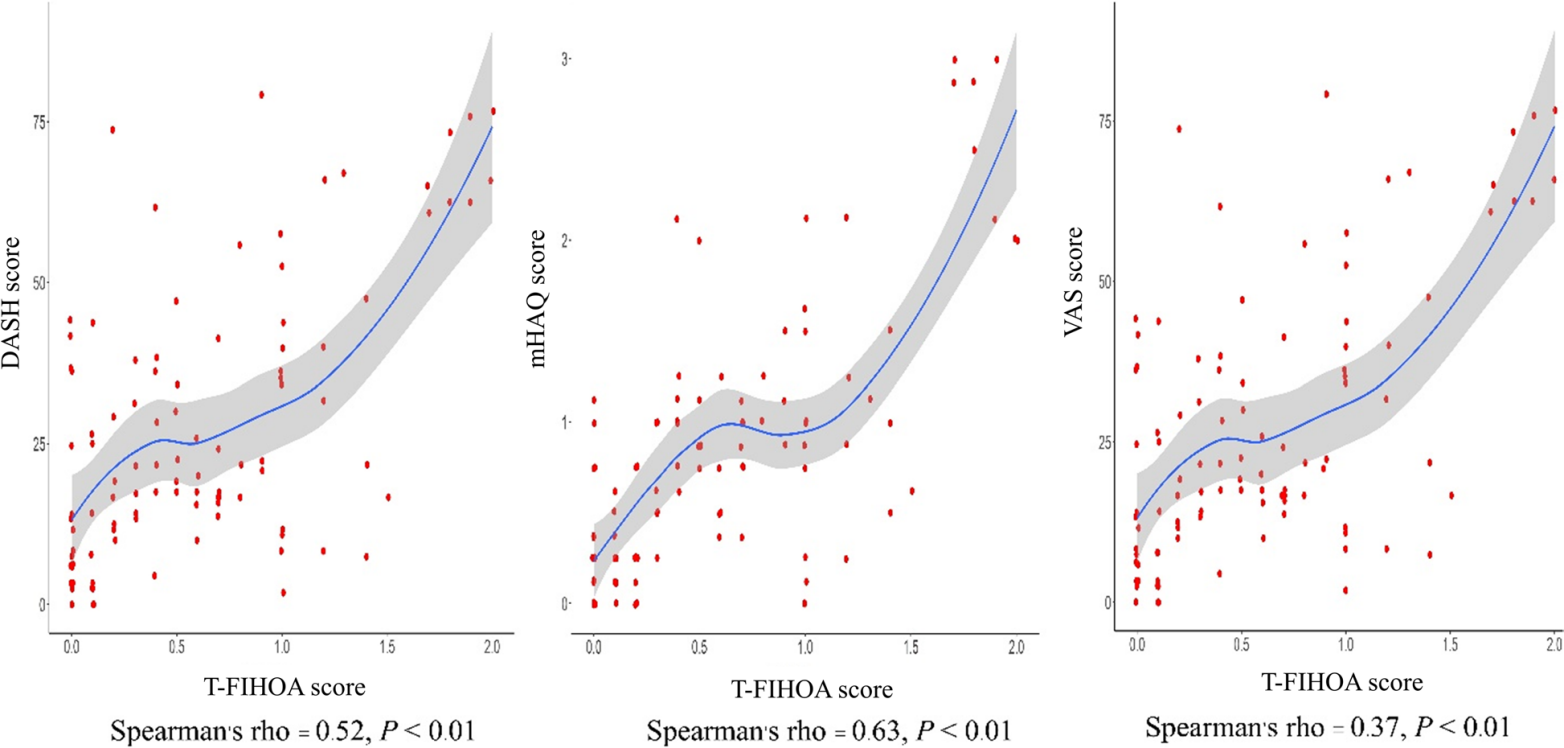
External construct validity of T-FIHOA compared to hand pain VAS, mHAQ, and DASH scores

The Spearman’s rho values for the correlations between the total T-FIHOA score, hand pain VAS, mHAQ, and DASH scores were calculated for external validity. There was a moderately significant direct correlation between the T-FIHOA and the hand pain VAS (Spearman’s rho = 0.37, *P* < 0.01) which was expected since the FIHOA is not a pain scale, and strong significant direct correlations between T-FIHOA and mHAQ (Spearman’s rho = 0.63, *P* < 0.01) and T-FIHOA and the DASH score (Spearman’s rho = 0.52, *P* < 0.01) (Fig. 2).

### Responsiveness

Symptomatic participants defined by a total T-FIHOA score ≥ 5 (*n* = 51) were included in the responsiveness analysis. Change in scores before the treatment and 4-week follow up were compared using the Wilcoxon signed-rank test. The effect size (ES) and standardized response mean (SRM) were calculated. ES was defined as mean change divided by the standard deviation (SD) and SRM was defined as mean changes divided by the SD of that change. The responsiveness was evaluated by comparing of ES and SRM between the T-FIHOA and other measurements. Results are shown in Table 5: ES and SRM of the T-FIHOA were 0.37 and 0.58 respectively.

**Table 5:** Responsiveness of T-FIHOA and other questionnaires in this study

Questionnaire	Pre-treatment Mean (SD)	Post-treatment Mean (SD)	Change Mean (SD)	*P* value	ES	SRM
T-FIHOA	7.5 (6.2)	5.2 (4.9)	2.3 (3.9)	< 0.01	−0.37	−0.58
mHAQ	1.0 (0.8)	0.8 (0.6)	0.2 (0.4)	< 0.01	−0.25	− 0.49
DASH	29.9 (22.4)	23.5 (17.3)	6.4 (11.3)	< 0.01	−0.29	−0.57
VAS score	63.7 (14.7)	33.9 (17.9)	29.8 (11.7)	< 0.01	−2.03	−2.54

## Discussion

This study aimed at developing a Thai version of the FIHOA, the T-FIHOA. After translation and cultural adaptation, a validation study was conducted to assessing test-retest reliability, internal consistency, and internal and external construct validity. The T-FIHOA had good reliability and validity in Thai patients with OA in the hand. The results from this study indicate that T-FIHOA is a valid and reliable questionnaire to evaluate functional disability in Thai-speaking patients with hand OA. Overall, the universal character of each item can easily be understood in any language and culture. The methodology used in this study for translation, cultural adaptation and validation was similar to that used for previous linguistic versions of FIHOA [9, 18–24].

The reliability results were good as indicated by Spearman’s rho and ICC values. The correlations between the 1st and 2nd completions of the T-FIHOA were excellent at each item and at the global score level, suggesting that this questionnaire has a good intra-observer reliability.

The T-FIHOA showed also a strong internal coherence. Cronbach alpha values were decreased when deleting items one after the other meaning that each item is useful and consistent. A factor analysis of T-FIHOA suggested that it is a unidimensional questionnaire. Loading factors were in adequation with the minimal requirements being all over the 0.5 cut-off value [29]. The results of this study were consistent with the original FIHOA by Dreiser et al. [8], and also the Norwegian and Japanese versions of the FIHOA [19, 22] which concluded it was a good tool for assessing hand OA functional impairment. The T-FIHOA is a unidimensional scale with good internal consistency as attested by Cronbach’s alphas values over 0.9 [30].

Thai validated versions of mHAQ and DASH measurements were used to assess the external consistency of the T-FIHOA. T-FIHOA strongly correlates with mHAQ and DASH scores, and less correlates with hand pain VAS which confirms its ability to capture hand-OA related dysfunction. Correlation with mHAQ were in line with other previously reported results (ranging from 0.57 to 0.73) [18–24, 27]. The correlation with DASH was slightly lower than with mHAQ probably because the DASH score assesses function of the upper limb, and not specifically that of hand. The correlation with the pain score was weaker which is consistent with the fact that the FIHOA does not assess pain intensity. However, there are discrepancies in correlations between FIHOA and pain scores among currently published studies [18, 22–24]. In this study, we used ES and SRM to assess the responsiveness. The T-FIHOA exhibited the highest ES and SRM values among all patients reported outcome measures, except for the VAS pain score. A possible explanation is that the T-FIHOA was developed as a hand OA-specific scale and may better detect loss of hand function than other non-hand specific assessment tools. The best responsiveness was observed with the VAS pain score since pain is often the most sensitive outcome on the short term in HOA, as previously shown in a clinical trial by Kvien [31]. Results were consistent with those of this clinical trial [31] and the validation study of the Japanese FIHOA [22].

Our study has some limitations. First, our population was only recruited from a tertiary care unit in the South of Thailand which may not reflect data in the overall Thai population. However, due to the simplicity of items and the socio-cultural similarity between Thai areas, we believe that our results are valid and of use for the entire Thai population. Second, this study examined the T-FIHOA only with educated patients because illiterate people would be unable to read the Thai alphabet and, particularly could not respond to item 9 which asks “Can you write for a long period of time?”

## Conclusions

The T-FIHOA showed good psychometric properties to assess hand OA-related functional disability in Thai people. Since it is now validated, and freely available for the community, it may and should be used by all practioners in daily practice hand OA, assessment, and by researchers in clinical trials or surveys.

## Data Availability

The datasets analyzed during the current study are available from the corresponding author on reasonable request.

## Abbreviations

FIHOA: Functional Index for Hand Osteoarthritis
T-FIHOA: Thai Functional Index for Hand Osteoarthritis
DASH: Disabilities of the Arm, Shoulder and Hand
mHAQ: Modified Health Assessment Questionnaire
VAS: Visual analogue scale
OA: Osteoarthirtis
ICC: Intraclass correlation coefficient
ES: Effect size
SRM: Standardized response mean
SD: Standard deviation
